# Impact of comorbidities in COPD clinical control criteria. The CLAVE study

**DOI:** 10.1186/s12890-023-02758-0

**Published:** 2024-01-02

**Authors:** Pere Almagro, Juan José Soler-Cataluña, Arturo Huerta, Diego González-Segura, Borja G. Cosío, on behalf of the CLAVE Study Investigators

**Affiliations:** 1 Multimorbidity Patients Unit. Internal Medicine Department, H. Mutua Terrassa University Hospital, Plaza del Doctor Robert, 5, 08221, Terrassa, Barcelona, Spain; 2 Department of Pneumology, Hospital Arnau de Vilanova-Lliria, Medicine Department, València University and CIBERES, Valencia, Spain; 3 Pulmonary and Critical Care Division, Emergency Department, Clínica Sagrada Família, Barcelona, Spain; 4 Medical Department, Chiesi SAU, Barcelona, Spain; 5 Department of Pneumology, H. Universitari Son Espases Hospital-IdISBa and CIBERES, Palma de Mallorca, Balearic Islands Spain

**Keywords:** COPD: Chronic obstructive pulmonary disease, CAT: COPD assessment test, Comorbidities, Clinical control criteria, Exacerbations, Charlson index

## Abstract

**Background:**

Chronic obstructive pulmonary disease (COPD) frequently coexists with other chronic diseases, namely comorbidities. They negatively impact prognosis, exacerbations and quality of life in COPD patients. However, no studies have been performed to explore the impact of these comorbidities on COPD clinical control criteria.

**Research question:**

Determine the relationship between individualized comorbidities and COPD clinical control criteria.

**Study design and methods:**

Observational, multicenter, cross-sectional study performed in Spain involving 4801 patients with severe COPD (< 50 predicted forced expiratory volume in the first second [FEV_1_%]). Clinical control criteria were defined by the combination of COPD assessment test (CAT) scores (≤16 vs ≥17) and exacerbations in the previous three months (none vs ≥1). Binary logistic regression adjusted by age and FEV_1_% was performed to identify comorbidities potentially associated with the lack of control of COPD. Secondary endpoints were the relationship between individualized comorbidities with COPD assessment test and exacerbations within the last three months.

**Results:**

Most frequent comorbidities were arterial hypertension (51.2%), dyslipidemia (36.0%), diabetes (24.9%), obstructive sleep apnea-hypopnea syndrome (14.9%), anxiety (14.1%), heart failure (11.6%), depression (11.8%), atrial fibrillation (11.5%), peripheral arterial vascular disease (10.4%) and ischemic heart disease (10.1%). After age and FEV_1_% adjustment, comorbidities related to lack of clinical control were cardiovascular diseases (heart failure, peripheral vascular disease and atrial fibrillation; *p* < 0.0001), psychologic disorders (anxiety and depression; all *p* < 0.0001), metabolic diseases (diabetes, arterial hypertension and abdominal obesity; all *p* < 0.001), sleep disorders (*p* < 0.0001), anemia (*p* = 0.015) and gastroesophageal reflux (*p* < 0.0001). These comorbidities were also related to previous exacerbations and COPD assessment test scores.

**Interpretation:**

Comorbidities are frequent in patients with severe COPD, negatively impacting COPD clinical control criteria. They are related to health-related quality of life measured by the COPD assessment test. Our results suggest that comorbidities should be investigated and treated in these patients to improve their clinical control.

**Take-home points:**

**Study question:** What is the impact of comorbidities on COPD clinical control criteria?

**Results:** Among 4801 patients with severe COPD (27.5% controlled and 72.5% uncontrolled), after adjustment by age and FEV_1_%, comorbidities related to lack of clinical control were cardiovascular diseases (heart failure, peripheral vascular disease and atrial fibrillation; *p* < 0.0001), psychologic disorders (anxiety and depression; *p* < 0.0001), metabolic diseases (diabetes, arterial hypertension and abdominal obesity; *p* < 0.001), obstructive sleep apnea-hypopnea syndrome (*p* < 0.0001), anaemia (*p* = 0.015) and gastroesophageal reflux (*p* < 0.0001), which were related to previous exacerbations and COPD assessment test scores.

**Interpretation:** Comorbidities are related to health-related quality of life measured by the COPD assessment test scores and history of exacerbations in the previous three months.

**Supplementary Information:**

The online version contains supplementary material available at 10.1186/s12890-023-02758-0.

According to the Global Initiative for Chronic Obstructive Lung Disease (GOLD) guidelines, chronic obstructive pulmonary disease (COPD) is a common, preventable, and treatable disease characterized by persistent respiratory symptoms and airflow limitation due to airway and/or alveolar abnormalities, usually caused by significant exposure to noxious particles or gases [1]. COPD has experienced a steady increase during the last decades, affecting to over 400 million people globally, becoming the third leading cause of death for chronic diseases, with 3.2 million patients dying due to this disease [2, 3]. Recent normative and guidelines consider COPD an heterogeneous disease in which prognosis and treatment include the severity of the airway obstruction, symptoms and risk of exacerbations [1, 4, 5]. A recent, dynamic definition of clinical control criteria in COPD has been proposed, combining the impact and the stability of the disease over time [6–8]. A low clinical impact is considered when patients meet three out of the four clinical variables: low dyspnea grade, no need for rescue treatment more than three times within the last week, clear sputum color, and daily physical activity ≥30 min each day. Clinical stability is defined when patients do not experience exacerbations within the previous three months and have a favorable perception of their health status. In a simple, alternative definition, patients are considered clinically controlled if they had a COPD assessment test (CAT) score of ≤16 and no exacerbations within the last three months [6, 9, 10]. This classification is usually used since it is similar to the one in the GOLD guidelines, although with different thresholds [1]. To classify symptoms, GOLD considers a cut-off point of ≥2, equivalent to the modified Medical Research Council (mMRC) dyspnea scale to a score of ≥10 points in CAT. However, several studies have shown that a score of ≥2 in mMRC corresponds better to a CAT score of ≥17, while an mMRC score of ≤1 is roughly equivalent to CAT scores of ≤10 [11, 12]. On the other hand, the presence of one or more exacerbations within the previous three months is usually assessed, since it can better capture the degree of control of patients in comparison to the exacerbation history during the last year [13]. The definition of clinical control used in our study has shown a good concordance with previous control criteria, sensitivity to change and is related to the risk of exacerbations and mortality [6, 8, 9, 14, 15].

COPD is also characterized by a systemic component, with an increased prevalence of concurrent chronic diseases. They are defined as comorbidities when describing the burden of several chronic illnesses coexisting with a particular disease of interest, in this case, COPD. By contrast, when the importance relies in the interaction between multiple chronic conditions in the same subject, multimorbidity seems to be a more appropriate concept, since the simultaneous combination of these chronic diseases affects differently the symptoms, prognosis, and treatment of an individual patient [16, 17]. The more frequent chronic disorders associated with COPD are cardiovascular, metabolic and psychological diseases. These comorbidities are often undiagnosed and hence undertreated [18]. Prospective studies performed on severe COPD patients show that virtually all of them had at least another chronic disease, and half of them had four or more comorbidities [19, 20]. These chronic diseases are often related to smoking, systemic inflammation, and ageing, but they occur at younger ages in patients with COPD than in the general population [21]. Comorbidities negatively impact the prognosis, symptoms, and quality of life in COPD patients, but to our knowledge, their impact on clinical control criteria has only been partially explored [16, 19, 20, 22].

The main objective of this study was to explore the impact of individualized comorbidities on COPD clinical control criteria and their relationship with the CAT and previous exacerbations.

## Study design and methods

### Study design

The CLAVE study was an observational, cross-sectional, multicenter cohort analysis involving patients with severe COPD in Spain. Details of the study protocol have been described elsewhere [6]. Briefly, participants included in this study were males and females aged ≥40 years, with a smoking history of ≥10 pack-year; predicted post-bronchodilator forced expiratory volume in the first second (FEV_1_%) of < 50%, and in chronic COPD treatment. Patients receiving oral corticosteroids or antibiotics for a recent COPD exacerbation were excluded. The study protocol was approved by the Research Ethics Committee of the Hospital Clinic of Barcelona (Spain), and procedures were performed in accordance with the Declaration of Helsinki.

### Endpoints and variables

The primary endpoint was the association between individualized comorbidities and the clinical control of COPD. Patients with a CAT score of ≤16 and no exacerbations within the last three months were considered clinically controlled subjects. The CAT is an 8-item questionnaire designed to evaluate Health-Related Quality of Life (HRQL) in COPD patients. Each item can be scored from 0 (no limitation) to 5 (very limited) [23]. Comorbidities were documented using the previously validated Charlson index, a standard scale with 19 chronic diseases graded for disease severity without age adjustment. Since all patients had COPD, which adds a point to this index, the minimum score was 1 point [24]. Additionally, comorbidity data was collected using a specific previously published questionnaire that included relevant pathologies in COPD, whether incorporated or not in the Charlson index [25]. The validated Spanish version of CAT, alongside the number of exacerbations in the past three months and the number of chronic treatments, were compared with each comorbidity for the secondary endpoints. In addition, the BODEX and CODEX indexes were also evaluated regarding COPD clinical control criteria.

### Statistical analyses

Quantitative variables were presented as mean and standard deviation (SD) if data followed a normal distribution, with comparisons performed with the Student’s t-test. Non-normally distributed variables were described as median and interquartile ranges (IQR: 25–75%) and analyzed with non-parametric test (Mann-Whitney U test). Qualitative variables were expressed as number and percentage and analyzed with the χ^2^ test or the Fisher exact test. A binary logistic regression was carried out to identify comorbidities potentially associated with COPD clinical control criteria after age and FEV_1_% adjustment. Odds ratio (OR) and 95% confidence interval (95% CI) were reported. Statistical significance was set at *p* < 0.05. Statistical analyses were performed with SAS version 9.4.

## Results

A total of 4801 patients were included. Of those, 1322 (27.5%) were considered as controlled, and 3479 (72.5%) as uncontrolled for COPD clinical criteria according to CAT scores (≤16 vs ≥17) and exacerbations in the previous three months (none vs ≥1). Most of them were male (82.2%) living in urban areas (64.7%) with a mean age of 69.6 ± 9.3 years, with more than half of the sample (51.7%) being above 70 years old. Most participants (75.7%) were former smokers with a mean of 50.8 ± 25.7 packs-years, and a mean predicted FEV_1_% of 39.0 ± 8.3. Most of them had moderate or low physical activity (43.2% and 40.4%, respectively) and had good treatment adherence (61.0%) (Table 1).

**Table 1:** Population characteristics according to COPD clinical control criteria

	Whole sample (*n* = 4801)	Patients with controlled COPD (*n* = 1322)	Patients with uncontrolled COPD (*n* = 3479)	*p*-value^a^
**Gender, n (%)**				
Male	3947 (82.2)	1103 (83.4)	2844 (81.7)	0.1722
Female	854 (17.8)	219 (16.6)	635 (18.3)	
**Age, years**				
Median (P25; P75)	70.0 (63.0; 76.0)	69.0 (63.0; 75.0)	70.0 (63.0; 77.0)	< 0.0001
**Active smoking, n (%)**				
Yes	1168 (24.3)	333 (25.2)	835 (24.0)	0.3915
No ^b^	3633 (75.7)	989 (74.8)	2644 (76.0)	
**Number of packs-year, n**^**c**^				
Median (P25; P75)	45.0 (33.0; 60.0)	45.0 (35.0; 60.0)	45.0 (32.3; 60.0)	0.4344
**Level of care, n (%)**^**d**^				
Primary health care	337 (7.1)	57 (4.4)	280 (8.1)	< 0.0001
Specialists	4426 (92.9)	1251 (95.6)	3175 (91.9)	
**Degree of physical activity (IPAQ), n (%)**^**e**^				
High	738 (16.5)	316 (26.0)	422 (12.9)	< 0.0001
Moderate	1937 (43.2)	626 (51.4)	1311 (40.1)	
Low or inactive	1811 (40.4)	275 (22.6)	1536 (47.0)	
**Post-bronchodilator FEV**_**1**_**%**				
Median (P25; P75)	41.0 (33.0; 46.0)	42.4 (35.2; 47.0)	40.0 (32.0; 46.0)	< 0.0001
**Degree of treatment adherence (TAI), n (%)**^**f**^				
Good	2767 (61.0)	850 (68.1)	1917 (58.3)	< 0.0001
Intermediate	887 (19.6)	242 (19.4)	645 (19.6)	
Bad	883 (19.5)	157 (12.6)	726 (22.1)	

^a^Mann-Whitney non-parametric U test was used to analyze differences between quantitative variables and the Chi-Square test for qualitative variables

^b^Ex-smokers: abstinence for at least the last six months

^c^Packs-year = [number of cigarettes per day * number of years] / 20

^d^*n* = 4763 patients with information about the level of care

^e^*n* = 4486 patients with answered IPAQ

^f^*n* = 4537 patients with answered TAI

**Fig. 1:**
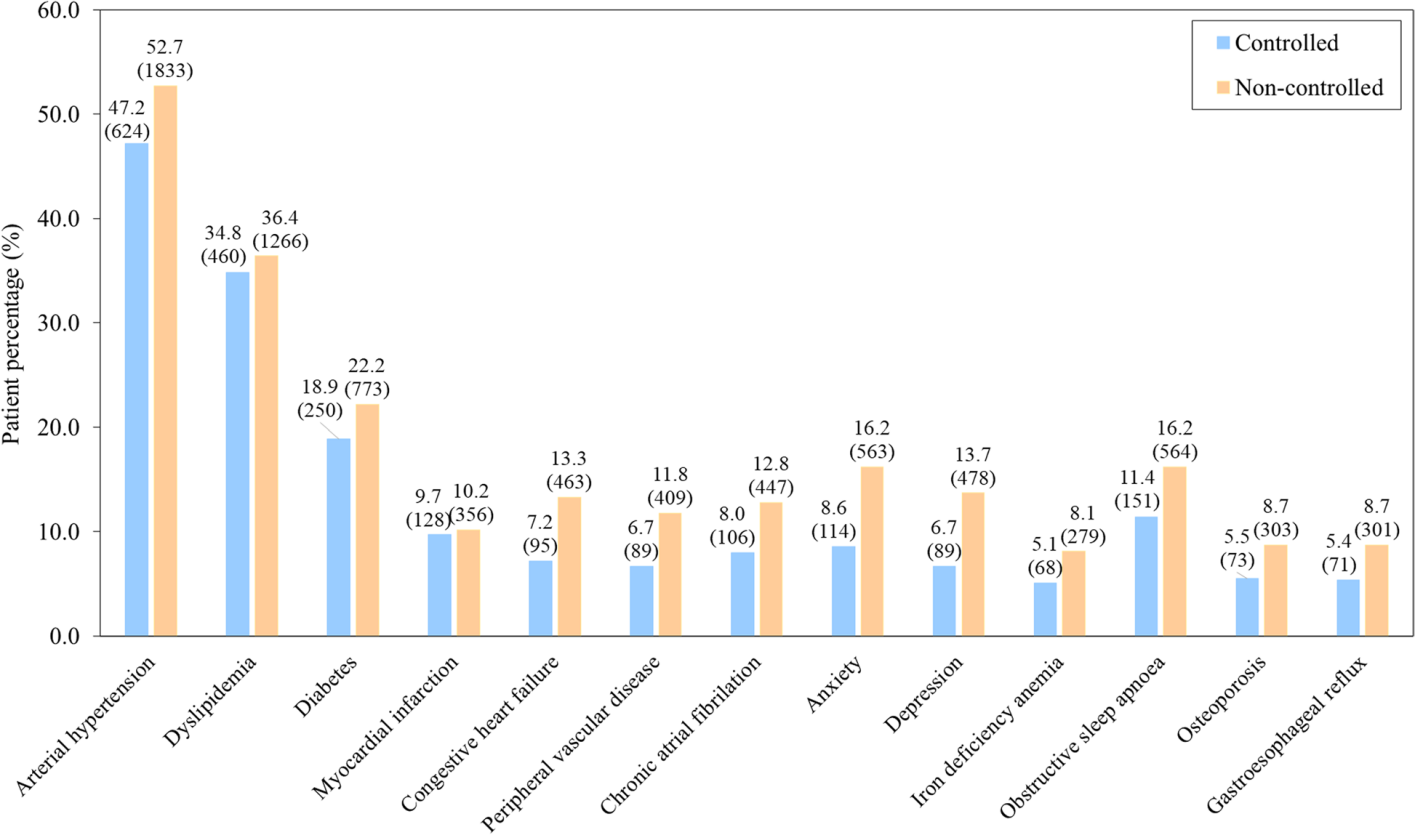
Prevalence of individualized comorbidities according to COPD clinical control criteria. Values are represented as percentage and number of patients

**Fig. 2:**
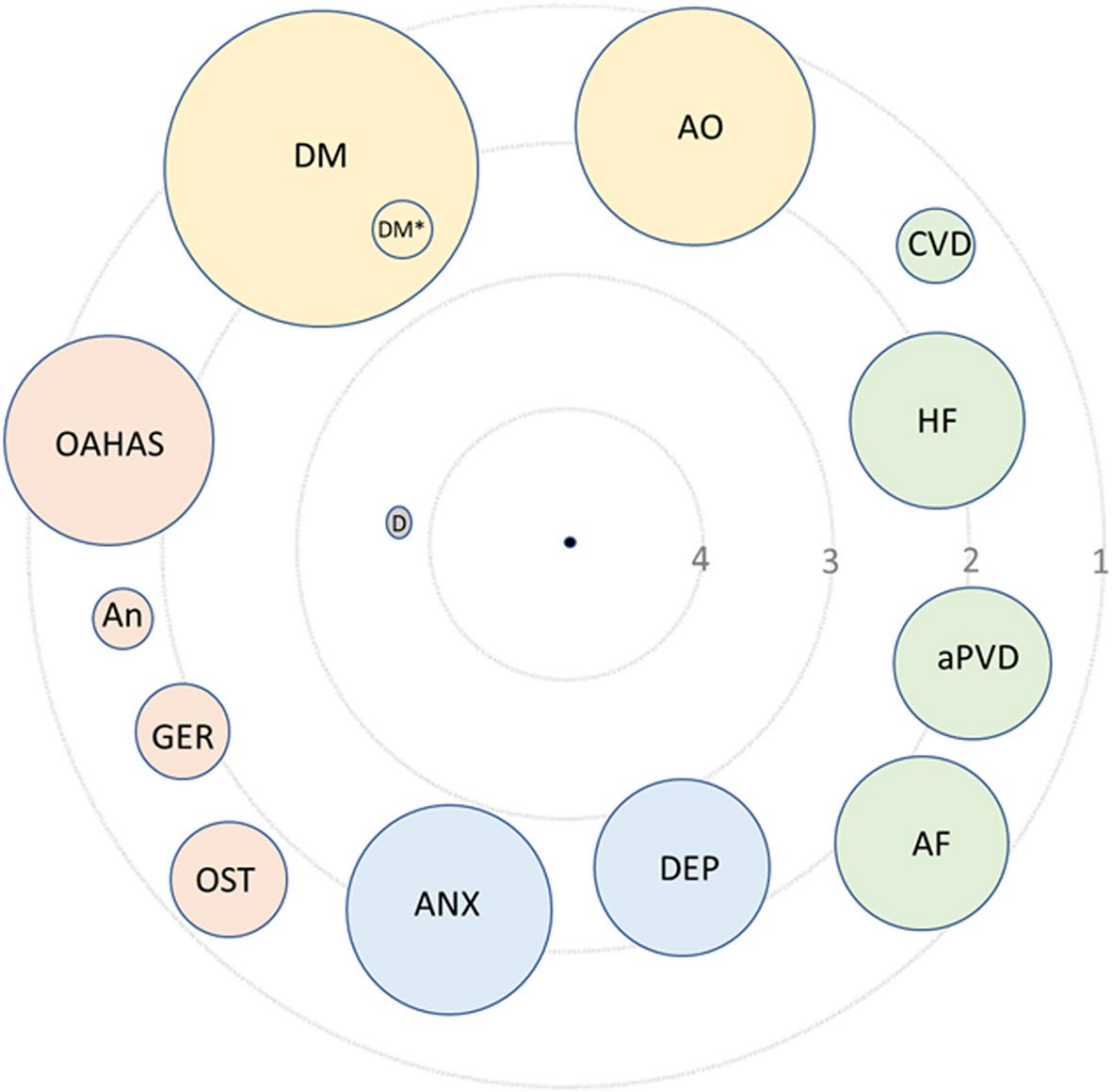
COPD comorbidome. Binary logistic regression adjusted by age and FEV_1_% to identify comorbidities potentially associated with the lack of control of COPD. The size of the circles represents the prevalence of individualized diseases, while the distance to the centre represents the odds ratio for diseases and COPD control (the closer the disease is to the center of the figure, the more negative it is for COPD control). The central black circle (center) represents the lack of control of COPD. Green circles: cardiovascular diseases. Yellow circles: metabolic diseases. Blue circles: psychologic disorders. Orange circles: other diseases. Grey circle: dementia. A.O.: abdominal obesity. CVD: cerebrovascular diseases. H.F.: heart failure. aPVD: arterial periferic vascular disease. A.F.: atrial fibrillation. DEP: depression. ANX: anxiety. OST: osteoporosis. GER: gastroesophageal reflux. AN: anaemia. OAHAS: obstructive apnoea-hypopnoea syndrome. D.M.: diabetes mellitus: D.M.*: diabetes mellitus with organ damage. D: dementia

The most frequent clusters of comorbidities were metabolic disorders (arterial hypertension [51.2%]; dyslipidemia [36.0%]; diabetes mellitus [24.9%]; abdominal obesity [15.8%]), cardiovascular diseases (myocardial infarction [10.1%]; heart failure [11.6%]; peripheral vascular disease [10.4%]; atrial fibrillation [11.5%]), and psychological disorders (anxiety [14.1%]; depression [11.8%]) (Tables 2 and 3) (Fig. 1). Higher scores in the non-age adjusted Charlson index were related to lack of COPD clinical control (1.90 [1.35] vs 2.28 [1.6]; *p* < 0.0001). Individualized comorbidities significantly related to COPD control are specified in Table 2 and Fig. 2, while comorbidities not included in the Charlson index are detailed in Table 3 and Fig. 2, respectively. In the adjusted analysis, the most relevant clusters of comorbidities associated with COPD clinical control criteria were metabolic, cardiovascular, and psychological disorders, alongside gastroesophageal reflux, non-ferropenic anemia, osteoporosis, obstructive sleep apnea-hypopnea syndrome (OSAHS) and dementia. Dementia had the highest impact (OR: 3.9; 95% CI: 1.4–10.8; *p* = 0.0102) but a lower prevalence (1%).

**Table 2:** Association between the presence of comorbidities according to the non-adjusted Charlson index and COPD clinical control criteria

	Univariate analysis				Multivariate analysis^a^; OR (CI 95%); *p*-value
	Total	Controlled COPD	Non-controlled COPD	*p*-value	
**Charlson index, n (%)**	4801 (100.0)	1322 (100.0)	3479 (100.0)		
Mean (SD)	2.17 (1.54)	1.90 (1.35)	2.28 (1.6)	< 0.0001	
Comorbidities included in the Charlson index, n (%)					
Myocardial infarction	484 (10.1)	128 (9.7)	356 (10.2)	0.5714	1.03 (0.83–1.28); *p* = 0.7968
Congestive heart failure	558 (11.6)	95 (7.2)	463 (13,3)	< 0.0001	1.83 (1.45–2.31); *p* < 0.0001
Peripheral vascular disease	498 (10.4)	89 (6.7)	409 (11.8)	< 0.0001	1.80(1.41–2.29); *p* < 0.0001
Cerebrovascular disease	239 (5.0)	51 (3.9)	188 (5.4)	0.0278	1.32 (0.96–1.82); *p* = 0.0856
Dementia	49 (1.0)	4 (0.3)	45 (1.3)	0.0023	3.85 (1.38–10.79); *p* = 0.0102
Pathology of connective tissue	73 (1.5)	14 (1.1)	59 (1.7)	0.1072	1.74 (0.96–3.14); *p* = 0.0664
Ulcer disease	191 (4.0)	41 (3.1)	150 (4.3)	0.0553	1.32 (0.93–1.88); *p* = 0.1239
Mild liver pathology	220 (4.6)	49 (3.7)	171 (4.9)	0.0736	1.42 (1.02–1.97); *p* = 0.0353
Diabetes	1023 (21.3)	250 (18.9)	773 (22.2)	0.0124	1.22 (1.04–1.44); *p* = 0.0138
Diabetes with organic lesion	173 (3.6)	24 (1.8)	149 (4.3)	< 0.0001	2.33 (1.50–3.61); *p* = 0.0002
Hemiplegia	18 (0.4)	3 (0.2)	15 (0.4)	0.4298^f^	1.86 (0.53–6.50); *p* = 0.3299
Renal pathology (moderate or severe)	219 (4.6)	49 (3.7)	170 (4.9)	0.0801	1.22 (0.88–1.70); *p* = 0.2297
Solid neoplasm without metastasis	448 (9.3)	103 (7.8)	345 (9.9)	0.0237	1.21 (0.96–1.52); *p* = 0.1134
Leukemia	18 (0.4)	3 (0.2)	15 (0.4)	0.4298^f^	1.77 (0.51–6.19); *p* = 0.3686
Malignant lymphoma	18 (0.4)	2 (0.2)	16 (0.5)	0.1834^f^	3.10 (0.71–13.52); *p* = 0.1324
Liver pathology (moderate or severe)	73 (1.5)	13 (1.0)	60 (1.7)	0.0608	1.89 (1.03–3.48); *p* = 0.0408
Solid metastasis	18 (0.4)	3 (0.2)	15 (0.4)	0.4298^f^	1.97 (0.56–6.87); *p* = 0.2880
AIDS	30 (0.6)	7 (0.5)	23 (0.7)	0.6052	1.46 (0.62–3.44); *p* = 0.3925

^a^Binary logistic regression adjusted by age and FEV_1_% to identify comorbidities potentially associated with the lack of control of COPD

**Table 3:** Association between the presence of comorbidities non-included in the Charlson index and COPD clinical control criteria

	Univariate analysis				Multivariate analysis^a^; OR (CI 95%); *p*-value
	Total	Controlled	Non-controlled	*p*	
Asthma	192 (4.0)	49 (3.7)	143 (4.1)	0.5235	1.23 (0.88–1.72); *p* = 0.2185
Sleep disturbance (OAHAS or equivalent)	715 (14.9)	151 (11.4)	564 (16.2)	< 0.0001	1.57 (1.29–1.91); *p* < 0.0001
Lung neoplasm	125 (2.6)	32 (2.4)	93 (2.7)	0.6234	1.08 (0.72–1.63); *p* = 0.7109
Sinus node disease	37 (0.8)	8 (0.6)	29 (0.8)	0.4188	1.24 (0.56–2.73); *p* = 0.6001
Arterial hypertension	2457 (51.2)	624 (47.2)	1833 (52.7)	0.0007	1.20 (1.05–1.37); *p* = 0.0076
Chronic atrial fibrillation	553 (11.5)	106 (8.0)	447 (12.8)	< 0.0001	1.56 (1.25–1.96); *p* = 0.0001
Atrio-ventricular block	50 (1.0)	11 (0.8)	39 (1.1)	0.3784	1.24 (0.63–2.43); *p* = 0.5408
Thromboembolic disease (PTE or DVT precedents)	106 (2.2)	26 (2.0)	80 (2.3)	0.4833	1.18 (0.75–1.85); *p* = 0.4836
Iron-deficiency anemia (Hgb < 13 g/l)	193 (4.0)	41 (3.1)	152 (4.4)	0.0458	1.30 (0.91–1.85); *p* = 0.1512
Other anemia (Hgb13 g/l)	154 (3.2)	27 (2.0)	127 (3.7)	0.0047	1.69 (1.11–2.59); *p* = 0.0152
Dyslipidemia	1726 (36.0)	460 (34.8)	1266 (36.4)	0.3039	1.08 (0.94–1.24); *p* = 0.2664
Abdominal obesity (men > 102 cm; women 88 cm)	757 (15.8)	173 (13.1)	584 (16.8)	0.0017	1.42 (1.18–1.71); *p* = 0.0002
Osteoporosis	376 (7.8)	73 (5.5)	303 (8.7)	0.0002	1.58 (1.21–2.06); *p* = 0.0008
Anxiety	677 (14.1)	114 (8.6)	563 (16.2)	< 0.0001	2.06 (1.66–2.55); *p* < 0.0001
Depression	567 (11.8)	89 (6.7)	478 (13.7)	< 0.0001	2.21 (1.74–2.80); *p* < 0.0001
Gastroesophageal reflux	372 (7.7)	71 (5.4)	301 (8.7)	0.0001	1.72 (1.32–2.25); *p* < 0.0001
Digestive malignancy	55 (1.1)	16 (1.2)	39 (1.1)	0.7951	0.87 (0.48–1.57); *p* = 0.6405

OAHAS: Obstructive apnoea-hypopnea syndrome

^a^Binary logistic regression adjusted by age and FEV_1_% to identify comorbidities potentially associated with the lack of control of COPD

The most relevant metabolic comorbidities in the adjusted analysis were diabetes with organic damage (OR: 2.3; 95% CI: 1.5–3.6), diabetes without organic lesion (OR: 1.2; 95% CI: 1.0–1.44), abdominal obesity (OR: 1.4; 95% CI: 1.2–1.7) and arterial hypertension (OR: 1.2; 95% CI: 1.1–1.4). Significant cardiovascular comorbidities were congestive heart failure (OR: 1.8; 95% CI: 1.45–2.3), peripheral vascular disease (OR: 1.8; 95% CI: 1.4–2.3) and chronic atrial fibrillation (OR: 1.6; 95% CI: 1.2–2.0). Regarding psychological disorders, both depression (OR: 2.2; 95% CI: 1.7–2.8) and anxiety (OR: 2.1; 95% CI: 1.7–2.5) were also associated with a lack of COPD control. Other significant comorbidities related with COPD control were non-ferropenic anemia (OR: 1.7; 95% CI: 1.1–2.6), osteoporosis (OR: 1.6; 95% CI: 1.2–2.1) and obstructive sleep apnea-hypopnea syndrome (OR: 1.6; 95% CI: 1.3–1.9) (Tables 2 and 3; Fig. 2).

**Fig. 3:**
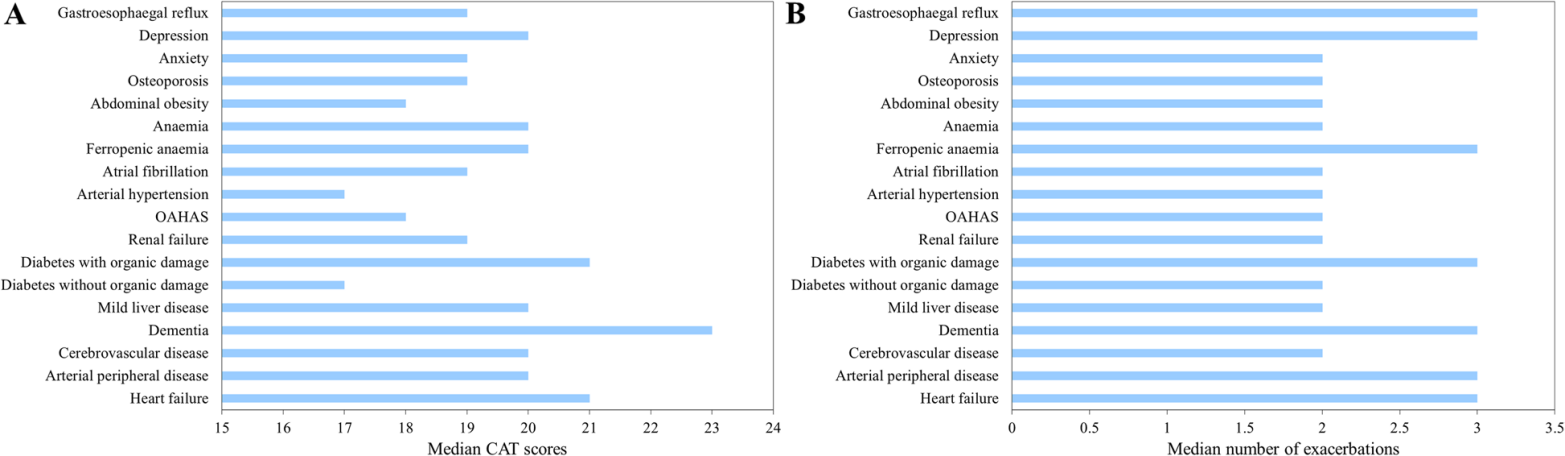
Median CAT scores (A) and number of exacerbations in the previous year (B) in individualized comorbidities

According to CAT scores, the most significant differences were observed for congestive heart failure, peripheral vascular disease, cerebrovascular disease, dementia, diabetes with and without organic lesion, iron-deficiency anemia and depression (eTable 1; Fig. 3). Several comorbidities were also related to a higher number of exacerbations within the last year (eTable 2; Fig. 3). The median number of chronic domiciliary treatments was increased in nearly all the comorbidities explored (eTable 3). The different inhaled treatments for COPD stratified according to the different comorbidities analyzed (included or not in the Charlson index) are detailed in the supplementary material. No differences were observed between the inhaled treatments and the different comorbidities, except for asthma, in which, as expected, there is less use of LABA+LAMA and more treatments with LABA+ICS and triple therapies (eTable 4 and eFigs. 1 and 2). Finally, scores in BODEX and CODEX indexes were higher in patients with uncontrolled COPD (eTable 5).

## Discussion

Our study confirms the high prevalence of comorbidities in patients with severe COPD and their impact on clinical control criteria, measured by CAT scores and history of previous exacerbations. This finding is relevant since it suggests that, at least in patients with advanced COPD, the concurrence of other chronic pathologies must be considered in the evaluation of clinical control criteria. Thus, two patients with the same respiratory condition can be classified as controlled or non-controlled depending on the presence of comorbidities. To our knowledge, this is the first study in which the relationship between detailed comorbidities and COPD clinical control criteria has been explored.

In the CHAIN cohort, in which changes in control status in 798 patients with COPD over long-term follow-up were described and the factors that were associated with longitudinal control patterns were analyzed, comorbidities assessed with the Charlson index were significantly associated with a lack of clinical control. However, the impact of individualized chronic diseases was not analyzed [8]. The mean values of the Charlson index in our study were higher than those reported in the CHAIN cohort, probably for the differences in mean age (69.6 vs 65.7 years) and severity of airway obstruction (mean FEV_1_%: 39 vs 60.2). Notably, mean CAT scores were also higher in our study (17.3 vs 12.6). On the contrary, no differences were observed in another study performed on 267 patients between the age-adjusted Charlson index and clinical control criteria [7, 15].

Nearly all patients with severe COPD had at least another concurrent chronic disease. Vanfleteren *et al.* identified in an observational study performed in a pulmonary rehabilitation program that 97.7% out of 213 patients had either one or more comorbidities, with 53.5% of them having at least four of these conditions [19]. These results are similar to those reported in a cohort of 606 COPD patients hospitalized by acute exacerbation [20]. Worthy of note, many of the most frequent comorbidities collected in these studies, such as arterial hypertension, psychologic disorders, arrhythmias, obesity or anemia, were not included in the Charlson index. Additionally, since concurrent chronic diseases in COPD are closely related to ageing and pulmonary function impairment, the impact of comorbidities in our study was analyzed after age and FEV_1_% had been adjusted.

Similarly, to previous publications, the most prevalent chronic diseases in our cohort are metabolic disorders such as arterial hypertension (51.2%), dyslipidemia (36.0%), diabetes (24.9%) and abdominal obesity (15.8%) [18–20, 26–29]. Their combination represent the metabolic syndrome, a strong predictor of the risk of cardiovascular events [30]. Metabolic syndrome is more frequent in COPD patients than in the general population. It is also related to higher levels of dyspnea, lower effort capacity measured as 6 minutes walking test (6MWD), higher CAT scores and more frequent exacerbations [31–33]. In previous studies, all individual components of metabolic syndrome were more prevalent in COPD, even after their adjustment with age, gender or socioeconomic level [34–37]. In our study, arterial hypertension, diabetes and abdominal obesity were negatively associated with clinical control criteria. Arterial hypertension and diabetes were previously associated with higher levels of dyspnea, and reduced 6MWD, while the relation between physical activity and abdominal obesity remained controversial [38].

Cardiovascular diseases are closely related to COPD. These conditions frequently concur in the same subjects, and their joint prevalence exceeds that expected for shared risk factors like smoking, ageing or low-grade systemic inflammation, among others [39]. The prevalence of heart failure is two-fold increased in COPD compared with the general population, although it varies largely among different design studies and analyzed populations [33, 40, 41]. In our study, 11.6% of the patients had a previous diagnosis of heart failure, with a two-fold adjusted risk for lack of clinical control criteria. This is not surprising since it is known that heart failure worsens the quality of life and increases the risk of exacerbations in COPD patients [25, 42, 43]. A similar prevalence and relation with non-controlled patients were observed for arterial peripheral vascular disease and chronic atrial fibrillation. Both disorders have been previously related to a lower quality of life and risk of exacerbations in COPD [44–46].

Depression and anxiety disorders are two to three times more likely in people with chronic diseases, including COPD, than those without chronic physical conditions [47, 48]. Both diseases are related to worse scores in COPD quality of life questionnaires, including CAT, and to a higher risk of exacerbations [45, 46, 49, 50]. In our cohort, depression and anxiety prevalence reached figures of 11.8 and 14.1%, respectively, and are two-fold more frequent in uncontrolled patients. Non-ferropenic anemia, sleep disorders (OAHS) and gastroesophageal reflux were also more frequent in patients with non-controlled COPD criteria. All of them were previously related to HRQL and exacerbations in different cohorts [45, 46, 51].

Finally, the comorbidity with the most significant impact on the COPD clinical control was dementia, but its prevalence was very low. In our opinion, this was due to an unavoidable selection bias. Although the incidence of dementia increases in patients with COPD and is a strong predictor of impaired quality of life, patients with moderate-severe dementia are usually unable to perform a quality spirometry, so they were excluded in our study because the obstruction criteria could not be demonstrated [52].

Our study had several limitations. Firstly, it was a cross-sectional study performed in a single country, although the sample size was considerably larger than that of previous publications about COPD clinical control criteria. Secondly, chronic diseases were collected according to clinical history and physical examination, and therefore several disorders, especially the less severe ones, could be underdiagnosed. Nevertheless, comorbidities were collected in a prospective form using validated questionnaires, and their prevalence was similar to the ones reported in previous cohorts. Thirdly, our study focused on patients with severe airflow obstruction, so our results might be different in other populations.

## Interpretation

In conclusion, our data confirm the relevance of several comorbidities in COPD clinical control criteria, supporting the notion that these conditions should be carefully evaluated in future studies. Our study warrants the need for prospective studies about the treatment impact of these chronic diseases on COPD control.

### Supplementary Information

**Additional file 1.**

**Additional file 2.**

## Data Availability

All data generated or analyzed during this study are included in this published article (and its supplementary information files).

## Abbreviations

CAT: COPD assessment test
COPD: Chronic obstructive pulmonary disease
FEV_1_: Forced expiratory volume in the first second
FVC: Forced vital capacity
GOLD: Global Initiative for Chronic Obstructive Lung Disease
HRQL: Health-Related Quality of Life
mMRC: Modified Medical Research Council dyspnea scale
OAHAS: Obstructive apnoea-hypopnea syndrome
FEV_1_%: Predicted postbronchodilator forced expiratory volume in the first second
6MWD: 6-minute walk distance
