# Supplementary material for: Impact of comorbidities in COPD clinical control criteria. The CLAVE study
Source: BMC Pulm Med. 2024 Jan 2;24:6. doi: 10.1186/s12890-023-02758-0 (PMC10759491; doi:10.1186/s12890-023-02758-0)

**Table e1:** CAT scores and comorbidities

|  | **With comorbidity** | **Without comorbidity** | **p^1^** |
| --- | --- | --- | --- |
| **Comorbidities included in the Charlson index; median (interquartile range)** | | | |
| Myocardial infarction | 17.5 (12.0-24.0) | 17.0 (11.0-23.0) | 0.0261 |
| Congestive heart failure | 21.0 (14.0-26.0) | 16.0 (11.0-22.0) | <0.0001 |
| Peripheral vascular disease | 20.0 (14.0-26.0) | 16.0 (11.0-22.0) | <0.0001 |
| Cerebrovascular disease | 20.0 (13.0-26.0) | 16.0 (11.0-23.0) | <0.0001 |
| Dementia | 23.0 (17.0-27.0) | 17.0 (11.0-23.0) | <0.0001 |
| Pathology of connective tissue | 20.0 (14.0-25.0) | 17.0 (11.0-23.0) | 0.0254 |
| Ulcer disease | 20.0 (13.0-25.0) | 17.0 (11.0-23.0) | <0.0001 |
| Mild liver disease | 20.0 (13.0-25.0) | 17.0 (11.0-23.0) | 0.0005 |
| Diabetes | 17.0 (12.0-24.0) | 16.0 (11.0-22.0) | 0.0012 |
| Diabetes with organic lesion | 21.0 (15.0-28.0) | 16.0 (11.0-23.0) | <0.0001 |
| Hemiplegia | 21.5 (16.0-30.0) | 17.0 (11.0-23.0) | 0.0175 |
| Renal pathology (moderate or severe) | 19.0 (13.0-27.0) | 17.0 (11.0-23.0) | <0.0001 |
| Solid neoplasm without metastasis | 18.0 (12.0-24.0) | 17.0 (11.0-23.0) | 0.0123 |
| Leukemia | 20.0 (16.0-32.0) | 17.0 (11.0-23.0) | 0.0167 |
| **Comorbidities non-included in the Charlson index; median (interquartile range)** | | | |
| Sleep disturbance (OSAHS or equivalent) | 18.0 (12.0-24.0) | 16.0 (11.0-23.0) | <0.0001 |
| Arterial hypertension | 17.0 (12.0-24.0) | 16.0 (11.0-22.0) | <0.0001 |
| Chronic atrial fibrillation | 19.0 (13.0-25.0) | 16.0 (11.0-23.0) | <0.0001 |
| Iron-deficiency anemia (Hgb <13 g/l) | 20.0 (15.0-26.0) | 16.0 (11.0-23.0) | <0.0001 |
| Other anemia (Hgb <13 g/l) | 20.0 (15.0-28.0) | 17.0 (11.0-23.0) | <0.0001 |
| Abdominal obesity (men >102 cm; women 88 cm) | 18.0 (12.0-24.0) | 16.0 (11.0-23.0) | <0.0001 |
| Osteoporosis | 19.0 (14.0-25.0) | 16.0 (11.0-23.0) | <0.0001 |
| Anxiety | 19.0 (13.0-25.0) | 16.0 (11.0-22.0) | <0.0001 |
| Depression | 20.0 (14.0-27.0) | 16.0 (11.0-22.0) | <0.0001 |
| Gastroesophageal reflux | 19.0 (13.0-25.0) | 16.0 (11.0-23.0) | <0.0001 |

^1^Non-parametric Mann-Whitney U test

OSAHS: Obstructive sleep apnea-hypopnea syndrome

Abdominal obesity (men >102 cm, women > 88 cm)

Anemia (Hgb <13 g/l)

**Table e2:** Comorbidities related to exacerbations within the last year.

|  | **With comorbidity** | **Without comorbidity** | **p^1^** |
| --- | --- | --- | --- |
| **Comorbidities included in the Charlson index; median (interquartile range)** | | | |
| Congestive heart failure | 3.0 (1.0-5.0) | 2.0 (1.0-3.0) | <0.0001 |
| Peripheral vascular disease | 3.0 (1.0-4.0) | 2.0 (1.0-3.0) | <0.0001 |
| Cerebrovascular disease | 2.0 (1.0-4.0) | 2.0 (1.0-3.0) | 0.0012 |
| Dementia | 3.0 (2.0-6.0) | 2.0 (1.0-3.0) | 0.0002 |
| Chronic pulmonary disease | 2.0 (1.0-3.0) | - | - |
| Ulcer disease | 2.0 (1.0-4.0) | 2.0 (1.0-3.0) | 0.0073 |
| Mild liver pathology | 2.0 (1.0-4.0) | 2.0 (1.0-3.0) | 0.0081 |
| Diabetes without lesion | 2.0 (1.0-4.0) | 2.0 (1.0-3.0) | 0.0004 |
| Diabetes with organic lesion | 3.0 (1.0-5.0) | 2.0 (1.0-3.0) | <0.0001 |
| Renal pathology (moderate or severe) | 2.0 (1.0-4.0) | 2.0 (1.0-3.0) | 0.0055 |
| Leukemia | 4.0 (2.0-5.0) | 2.0 (1.0-3.0) | 0.0091 |
| **Comorbidities non-included in the Charlson index; median (interquartile range)** | | | |
| Asthma | 2.0 (1.0-4.0) | 2.0 (1.0-3.0) | 0.0018 |
| Sleep disturbance (OSAHS or equivalent) | 2.0 (1.0-4.0) | 2.0 (1.0-3.0) | 0.0220 |
| Sinus node disease | 3.0 (1.0-5.0) | 2.0 (1.0-3.0) | 0.0290 |
| Arterial hypertension | 2.0 (1.0-4.0) | 2.0 (1.0-3.0) | <0.0001 |
| Chronic atrial fibrillation | 2.0 (1.0-4.0) | 2.0 (1.0-3.0) | <0.0001 |
| Thromboembolic disease (PTE or DVT precedents) | 3.0 (1.0-4.0) | 2.0 (1.0-3.0) | 0.0010 |
| Iron-deficiency anemia (Hgb <13 g/l) | 3.0 (1.0-4.5) | 2.0 (1.0-3.0) | <0.0001 |
| Other anemia (Hgb <13 g/l) | 2.0 (1.0-4.0) | 2.0 (1.0-3.0) | 0.0040 |
| Dyslipidemia | 2.0 (1.0-4.0) | 2.0 (1.0-3.0) | 0.0431 |
| Abdominal obesity (men >102 cm; women <88 cm) | 2.0 (1.0-4.0) | 2.0 (1.0-3.0) | 0.0439 |
| Osteoporosis | 2.0 (1.0-4.0) | 2.0 (1.0-3.0) | <0.0001 |
| Anxiety | 2.0 (1.0-4.0) | 2.0 (1.0-3.0) | <0.0001 |
| Depression | 3.0 (1.0-5.0) | 2.0 (1.0-3.0) | <0.0001 |
| Gastroesophageal reflux | 3.0 (1.0-5.0) | 2.0 (1.0-3.0) | <0.0001 |

^1^Non-parametric Mann-Whitney U test

OSAHS: Obstructive sleep apnea-hypopnea syndrome

Abdominal obesity (men >102 cm, women > 88 cm)

Anemia (Hgb <13 g/l)

Thromboembolic disease (PTE or DVT history)

**Table e3:** Number of chronic domiciliary treatments according to comorbidities described by the Charlson index and other reported comorbidities.

|  | **With comorbidity** | **Without comorbidity** | **p^1^** |
| --- | --- | --- | --- |
| **Comorbidities included in the Charlson index, median (interquartile range)** | | | |
| Myocardial infarction | 9.0 (6.0-11.0) | 5.0 (3.0-8.0) | <0.0001 |
| Congestive heart failure | 8.0 (6.0-11.0) | 5.0 (3.0-7.0) | <0.0001 |
| Peripheral vascular disease | 7.0 (5.0-10.0) | 5.0 (3.0-8.0) | <0.0001 |
| Cerebrovascular disease | 8.0 (6.0-11.0) | 5.0 (3.0-8.0) | <0.0001 |
| Dementia | 8.0 (6.0-10.0) | 5.0 (3.0-8.0) | <0.0001 |
| Chronic lung disease | 5.0 (3.0-8.0) | - | - |
| Pathology of connective tissue | 7.0 (5.0-10.0) | 5.0 (3.0-8.0) | <0.0001 |
| Ulcer disease | 6.0 (4.0-9.0) | 5.0 (3.0-8.0) | 0.0002 |
| Mild liver pathology | 6.0 (4.0-10.0) | 5.0 (3.0-8.0) | <0.0001 |
| Diabetes | 7.0 (5.0-10.0) | 5.0 (3.0-7.0) | <0.0001 |
| Diabetes with organic lesion | 9.0 (6.0-11.0) | 5.0 (3.0-8.0) | <0.0001 |
| Hemiplegia | 8.5 (5.0-11.0) | 5.0 (3.0-8.0) | 0.0087 |
| Renal pathology (moderate or severe) | 9.0 (7.0-12.0) | 5.0 (3.0-8.0) | <0.0001 |
| Solid neoplasm without metastasis | 6.0 (4.0-9.5) | 5.0 (3.0-8.0) | <0.0001 |
| Leukemia | 8.0 (6.0-11.0) | 5.0 (3.0-8.0) | 0.0014 |
| Malignant lymphoma | 7.0 (6.0-9.0) | 5.0 (3.0-8.0) | 0.0098 |
| Liver pathology (moderate or severe) | 6.0 (3.0-8.0) | 5.0 (3.0-8.0) | 0.5009 |
| Solid metastasis | 7.5 (4.0-9.0) | 5.0 (3.0-8.0) | 0.1206 |
| AIDS | 7.0 (6.0-10.0) | 5.0 (3.0-8.0) | 0.0018 |
| **Comorbidities not-included in the Charlson index median (interquartile range)** | | | |
| Asthma | 6.0 (3.0-9.0) | 5.0 (3.0-8.0) | 0.1692 |
| Sleep disturbance (OSAHS or equivalent) | 7.0 (4.0-10.0) | 5.0 (3.0-8.0) | <0.0001 |
| Lung neoplasm | 6.0 (3.0-9.0) | 5.0 (3.0-8.0) | 0.3904 |
| Sinus node disease | 7.0 (6.0-10.0) | 5.0 (3.0-8.0) | 0.0005 |
| Arterial hypertension | 6.0 (4.0-9.0) | 4.0 (2.0-6.0) | <0.0001 |
| Chronic atrial fibrillation | 8.0 (6.0-11.0) | 5.0 (3.0-8.0) | <0.0001 |
| Atrio-ventricular block | 7.0 (5.0-10.0) | 5.0 (3.0-8.0) | 0.0003 |
| Thromboembolic disease (PTE or DVT precedents) | 6.0 (4.0-10.0) | 5.0 (3.0-8.0) | 0.0014 |
| Iron-deficiency anemia (Hgb <13 g/l) | 9.0 (6.0-11.0) | 5.0 (3.0-8.0) | <0.0001 |
| Other anemia (Hgb <13 g/l) | 10.0 (7.0-12.0) | 5.0 (3.0-8.0) | <0.0001 |
| Dyslipidemia | 7.0 (4.0-10.0) | 4.0 (2.0-7.0) | <0.0001 |
| Abdominal obesity (men >102 cm, women 88 cm) | 7.0 (4.0-10.0) | 5.0 (3.0-8.0) | <0.0001 |
| Osteoporosis | 7.0 (4.0-10.0) | 5.0 (3.0-8.0) | <0.0001 |
| Anxiety | 6.0 (4.0-9.0) | 5.0 (3.0-8.0) | <0.0001 |
| Depression | 7.0 (5.0-10.0) | 5.0 (3.0-8.0) | <0.0001 |
| Gastroesophageal reflux | 6.0 (4.0-9.0) | 5.0 (3.0-8.0) | <0.0001 |
| Digestive malignancy | 6.0 (4.0-10.0) | 5.0 (3.0-8.0) | 0.0383 |

^1^Non-parametric Mann-Whitney U test

OSAHS: Obstructive sleep apnea-hypopnea syndrome

**Table e4:** Comorbidities and type of treatment.

|  | **Monotherapy: LABA** | **Monotherapy: LAMA** | **Other monotherapies** | **Dual combinations: LABA/LAMA** | **Dual combinations: LABA/ICS** | **Other dual combinations** | **Triple therapies** | **Rescue only** | **TOTAL** |
| --- | --- | --- | --- | --- | --- | --- | --- | --- | --- |
| **Comorbidities included in the Charlson index, n (%)** | | | | | | | | | |
| **Myocardial infarction** | 2 (0.4) | 13 (2.7) | 4 (0.8) | 170 (35.3) | 47 (9.8) | 3 (0.6) | 238 (49.4) | 5 (1.0) | 482 (100.0) |
| **Congestive heart failure** | 4 (0.7) | 24 (4.3) | 7 (1.3) | 163 (29.4) | 57 (10.3) | 2 (0.4) | 288 (51.9) | 10 (1.8) | 555 (100.0) |
| **Peripheral vascular disease** | 9 (1.8) | 17 (3.4) | 7 (1.4) | 147 (29.7) | 58 (11.7) | 3 (0.6) | 243 (49.1) | 11 (2.2) | 495 (100.0) |
| **Cerebrovascular disease** | 2 (0.8) | 11 (4.6) | 5 (2.1) | 81 (34.2) | 26 (11.0) | 0 (0.0) | 109 (46.0) | 3 (1.3) | 237 (100.0) |
| **Dementia** | 1 (2.1) | 2 (4.2) | 1 (2.1) | 15 (31.3) | 5 (10.4) | 0 (0.0) | 24 (50.0) | 0 (0.0) | 48 (100.0) |
| **Chronic pulmonary disease** | 41 (0.9) | 154 (3.2) | 41 (0.9) | 1589 (33.3) | 493 (10.3) | 16 (0.3) | 2402 (50.3) | 42 (0.9) | 4778 (100.0) |
| **Pathology of connective tissue** | 6 (8.2) | 2 (2.7) | 0 (0.0) | 25 (34.3) | 6 (8.2) | 1 (1.4) | 33 (45.2) | 0 (0.0) | 73 (100.0) |
| **Ulcer disease** | 3 (1.6) | 9 (4.8) | 2 (1.1) | 62 (33.2) | 21 (11.2) | 1 (0.5) | 86 (46.0) | 3 (1.6) | 187 (100.0) |
| **Mild liver disease** | 2 (0.9) | 10 (4.6) | 1 (0.5) | 71 (32.6) | 28 (12.8) | 0 (0.0) | 103 (47.3) | 3 (1.4) | 218 (100.0) |
| **Diabetes** | 7 (0.7) | 43 (4.2) | 9 (0.9) | 318 (31.3) | 116 (11.4) | 6 (0.6) | 510 (50.2) | 8 (0.8) | 1017 (100.0) |
| **Diabetes with organic lesion** | 3 (1.8) | 6 (3.5) | 4 (2.3) | 50 (29.2) | 24 (14.0) | 0 (0.0) | 78 (45.6) | 6 (3.5) | 171 (100.0) |
| **Hemiplegia** | 1 (5.9) | 0 (0.0) | 2 (11.8) | 4 (23.5) | 1 (5.9) | 0 (0.0) | 9 (52.9) | 0 (0.0) | 17 (100.0) |
| **Renal pathology (moderate or severe)** | 2 (0.9) | 10 (4.6) | 3 (1.4) | 59 (27.2) | 27 (12.4) | 1 (0.5) | 114 (52.5) | 1 (0.5) | 217 (100.0) |
| **Solid neoplasms without metastasis** | 0 (0.0) | 15 (3.4) | 6 (1.4) | 140 (31.4) | 37 (8.3) | 0 (0.0) | 246 (55.2) | 2 (0.5) | 446 (100.0) |
| **Leukemias** | 0 (0.0) | 1 (5.6) | 0 (0.0) | 6 (33.3) | 2 (11.1) | 0 (0.0) | 9 (50.0) | 0 (0.0) | 18 (100.0) |
| **Malignant lymphoma** | 0 (0.0) | 0 (0.0) | 0 (0.0) | 5 (27.8) | 2 (11.1) | 0 (0.0) | 11 (61.1) | 0 (0.0) | 18 (100.0) |
| **Liver pathology (moderate or severe)** | 1 (1.4) | 3 (4.1) | 1 (1.4) | 30 (41.1) | 5 (6.9) | 0 (0.0) | 29 (39.7) | 4 (5.5) | 73 (100.0) |
| **Solid metastasis** | 0 (0.0) | 1 (5.6) | 1 (5.6) | 4 (22.2) | 2 (11.1) | 0 (0.0) | 10 (55.6) | 0 (0.0) | 18 (100.0) |
| **AIDS** | 0 (0.0) | 0 (0.0) | 0 (0.0) | 10 (33.3) | 3 (10.0) | 0 (0.0) | 17 (56.7) | 0 (0.0) | 30 (100.0) |
| **Comorbidities not included in the Charlson index, n (%)** | | | | | | | | | |
| **Asthma** | 3 (1.6) | 5 (2.6) | 5 (2.6) | 22 (11.6) | 42 (22.1) | 2 (1.1) | 110 (57.9) | 1 (0.5) | 190 (100.0) |
| **Sleep disturbance (OSAHS or equivalent)** | 3 (0.4) | 24 (3.4) | 5 (0.7) | 215 (30.2) | 69 (9.7) | 4 (0.6) | 389 (54.6) | 4 (0.6) | 713 (100.0) |
| **Lung neoplasm** | 0 (0.0) | 5 (4.0) | 1 (0.8) | 50 (40.3) | 8 (6.5) | 0 (0.0) | 60 (48.4) | 0 (0.0) | 124 (100.0) |
| **Sinus node disease** | 0 (0.0) | 2 (5.4) | 2 (5.4) | 11 (29.7) | 0 (0.0) | 0 (0.0) | 20 (54.1) | 2 (5.4) | 37 (100.0) |
| **Arterial hypertension** | 26 (1.1) | 79 (3.2) | 21 (0.9) | 794 (32.5) | 260 (10.6) | 10 (0.4) | 1231 (50.4) | 23 (0.9) | 2444 (100.0) |
| **Chronic atrial fibrillation** | 5 (0.9) | 23 (4.2) | 8 (1.5) | 169 (30.8) | 61 (11.1) | 3 (0.6) | 270 (49.3) | 9 (1.6) | 548 (100.0) |
| **Atrio-ventricular block** | 1 (2.0) | 2 (4.0) | 0 (0.0) | 18 (36.0) | 6 (12.0) | 0 (0.0) | 22 (44.0) | 1 (2.0) | 50 (100.0) |
| **Thromboembolic disease (PTE or DVT precedents)** | 1 (0.9) | 3 (2.8) | 0 (0.0) | 41 (38.7) | 10 (9.4) | 0 (0.0) | 49 (46.2) | 2 (1.9) | 106 (100.0) |
| **Iron-deficiency anemia (Hgb <13 g/l)** | 1 (0.5) | 9 (4.7) | 2 (1.1) | 49 (25.7) | 18 (9.4) | 0 (0.0) | 107 (56.0) | 5 (2.6) | 191 (100.0) |
| **Other anemia (Hgb <13 g/l)** | 2 (1.3) | 3 (2.0) | 3 (2.0) | 33 (21.7) | 18 (11.8) | 1 (0.7) | 92 (60.5) | 0 (0.0) | 152 (100.0) |
| **Dyslipidemia** | 19 (1.1) | 66 (3.8) | 11 (0.6) | 535 (31.2) | 201 (11.7) | 4 (0.2) | 871 (50.7) | 10 (0.6) | 1717 (100.0) |
| **Abdominal obesity (men >102 cm, women 88 cm)** | 7 (0.9) | 21 (2.8) | 6 (0.8) | 226 (29.9) | 81 (10.7) | 4 (0.5) | 404 (53.5) | 6 (0.8) | 755 (100.0) |
| **Osteoporosis** | 2 (0.5) | 15 (4.0) | 5 (1.3) | 105 (27.9) | 49 (13) | 0 (0.0) | 196 (52.1) | 4 (1.1) | 376 (100.0) |
| **Anxiety** | 12 (1.8) | 16 (2.4) | 8 (1.2) | 191 (28.3) | 80 (11.8) | 4 (0.6) | 360 (53.3) | 5 (0.7) | 676 (100.0) |
| **Depression** | 7 (1.2) | 11 (2.0) | 4 (0.7) | 168 (29.7) | 60 (10.6) | 2 (0.4) | 309 (54.7) | 4 (0.7) | 565 (100.0) |
| **Gastroesophageal reflux** | 2 (0.5) | 19 (5.2) | 6 (1.6) | 121 (32.9) | 46 (12.5) | 0 (0.0) | 170 (46.2) | 4 (1.1) | 368 (100.0) |
| **Digestive malignancy** | 0 (0.0) | 2 (3.6) | 0 (0.0) | 19 (34.6) | 4 (7.3) | 0 (0.0) | 30 (54.6) | 0 (0.0) | 55 (100.0) |

OSAHS: Obstructive sleep apnea-hypopnea syndrome.

**Table e5:** BODEX/CODEX index values based upon the control of COPD.

|  | **Total sample**  **(n=4801)** | **Patients with controlled COPD**  **(n=1322)** | **Patients with uncontrolled COPD**  **(n=3479)** | **p^1^** |
| --- | --- | --- | --- | --- |
| **BODEX index, median (interquartile range)** | 4.0 (3.0-5.0) | 3.0 (2.0-4.0) | 4.0 (3.0-5.0) | <0.0001 |
| **Age-adjusted CODEX index, median (interquartile range)** | 4.0 (3.0-6.0) | 3.0 (3.0-4.0) | 5.0 (4.0-6.0) | <0.0001 |
| **CODEX index, median (interquartile range)** | 4.0 (3.0-5.0) | 3.0 (2.0-4.0) | 4.0 (3.0-5.0) | <0.0001 |

^1^Non-parametric Mann-Whitney U test

**Figure e1:** Inhaled treatments detailed by comorbidities included in the Charlson index.


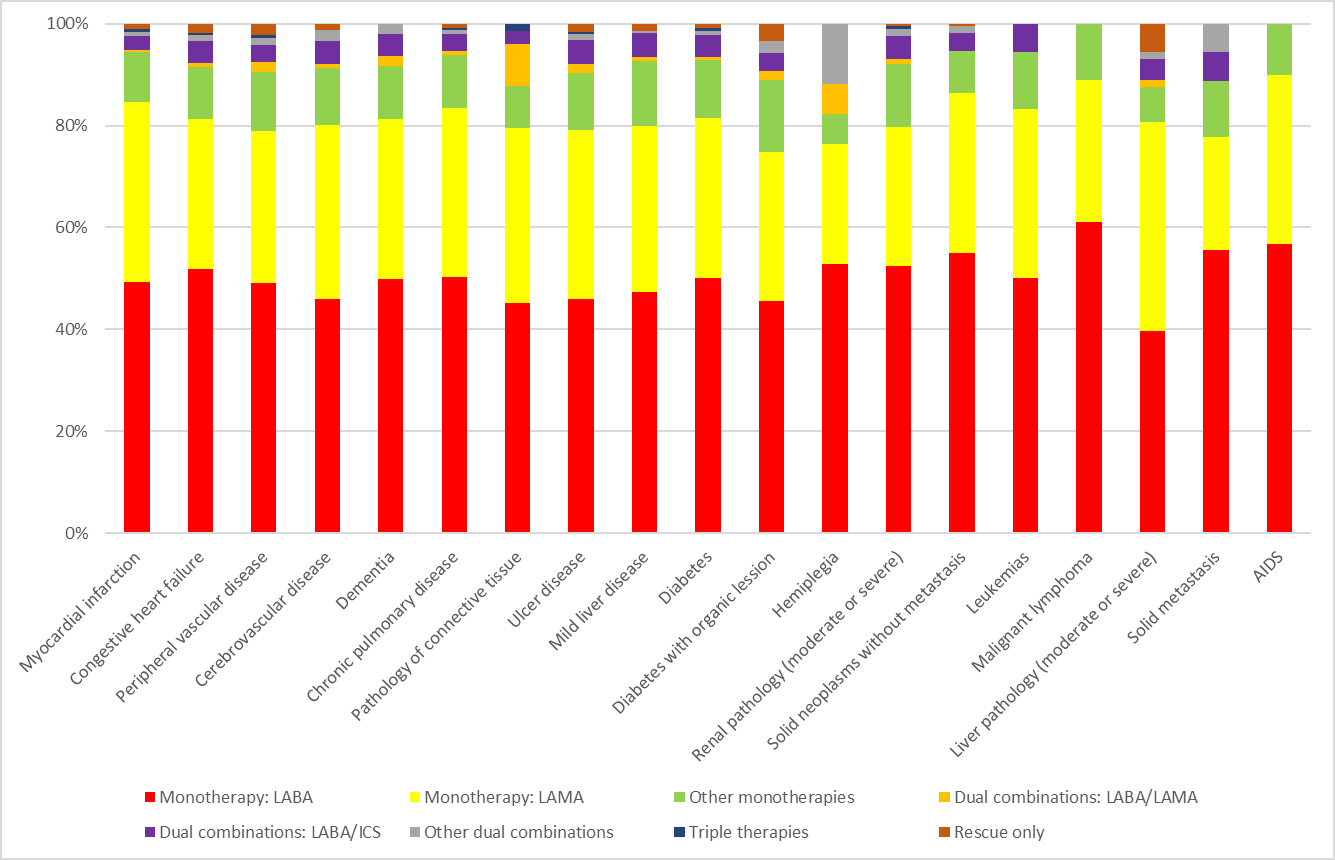


**Figure e2:** Inhaled treatments detailed by comorbidities not included in the Charlson index.


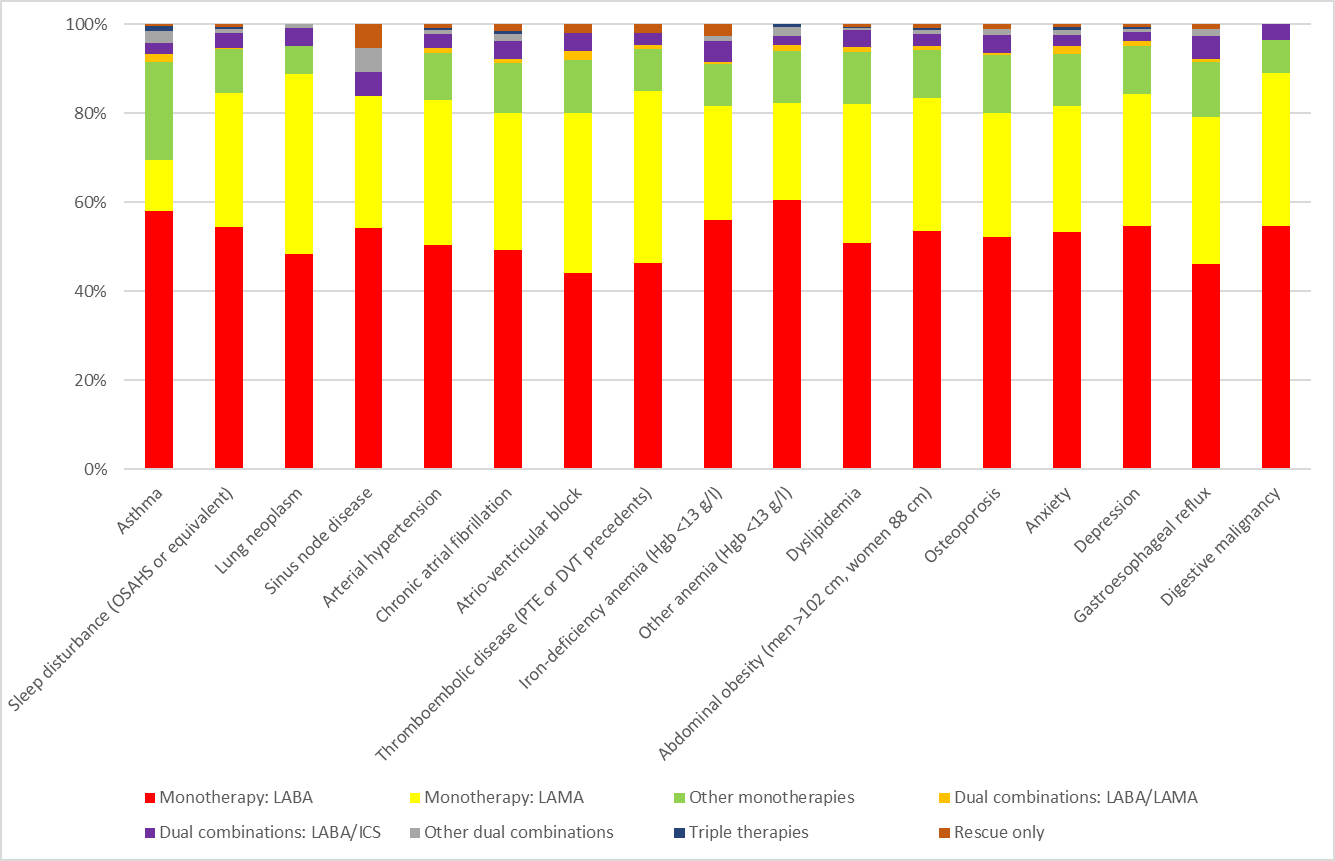

Supplement: Supplementary file 1 — Additional file 1. [file 12890_2023_2758_MOESM1_ESM.docx]
