# Supplementary material for: Impact of comorbidities in COPD clinical control criteria. The CLAVE study
Source: BMC Pulm Med. 2024 Jan 2;24:6. doi: 10.1186/s12890-023-02758-0 (PMC10759491; doi:10.1186/s12890-023-02758-0)
Supplement: Supplementary file 2 — Additional file 2. [file 12890_2023_2758_MOESM2_ESM.docx]

Participating investigators

Abel Pallarés Sanmartín, Abel Rodríguez Canal, Abelardo Montero Saez, Ada Luz Andreu Rodriguez, Adelaida Acuña Fernandez, Africa Alcorta Mesas, Agustín Valido Morales, Ainhoa Gomez, Aizea Mardones, Alba Calderón Pecellín, Alberto Díaz De Santiago, Alberto Herrejón Silvestre, Alberto Javier Nistal Rodriguez, Alberto Levy Naon, Alberto Saura Vinuesa, Alejandra Marin Arguedas, Alejandro Veres Racamonde, Alfonso Garcia Guisado, Alfredo Candela Blanes, Alicia Pérez Rodríguez, Alicia Ruiz Martín, Alvaro Flamarique Pascual, Alvaro Perez Gomez, Amaia Atenea Iridoy Zulet, Amaia Urrutia Gajate, Amparo Lloris Bayo, Amparo Romero Plaza, Ana Boldova Loscertales, ,Ana Botella Gregori, Ana Bustamante Ruiz, Ana Cobas Paz, Ana Fortuna Gutierrez, Ana García Peña, Ana Isabel Enríquez Rodríguez, Ana Kersul, Ana Maria Pueyo Bastida, Ana María Rodríguez Fernández, Ana Martín Medina, Ana Paramá Fontenla, Ana Paulina Gómez-Bastero, Ana Raño, Andres Rodero, Andrés Vilas Iglesias, Angel Blanco, Anibal Manuel Hernandez Gil, Anna Soler Sendra, Annie Navarro Rolon, Antoni Riba Blanch, Antonia Fuster Gomila, Antonia Mayol Mateu, ,Antonia Soto Venegas, Antonio Albaladejo Dávalos, Antonio Clavo, Antonio Drusetta Llahues, Antonio Eduardo Delgado Martín, Antonio Fernández Rodríguez, Antonio Jose Madueño Caro, Antonio Manuel Pérez Pernández, Antonio Muiño Miguez, Antonio Pereira Vega, Antonio Ruiz Peña, Antonio Sancho Muñoz, Araceli Abad Fernández, Araceli Fernandez Revuelta, Armando González López, Armando Larnia Sanchez, Arturo Huerta, Astrid Crespo Lessmann, Aurelio Arnedillo Muñoz, Ayman Soudan Barakat, Bartolome Poyato Gonzalez, Beatriz Abascal Bolado, Beatriz Arias Arcos, Beatriz Morales Chacón, Begoña Fernandez Infante, Begoña Ponce Buj, Belen Lopez-Muñiz, Belen Navas Bueno, Belen Orosa Bertol, Berta Avilés Huertas, Blanca Hortal Reina, Blas Rojo Moreno-Arrones, Borja Garcia-Cosio Piqueras, Carlos Alberto Názara Otero, Carlos Disdier, Carlos Javier Carpio, Carlos José Álvarez Martínez, Carlos Leon Rodriguez, Carlos Martinez Rivera, Carlos Peñalver Mellado, Carlos Soler Portmann, Carlos Vilariño Pombo, Carme Agusti, Carmen Abeledo, Carmen Angela Centeno Clemente, Carmen Elena Calvo Rodriguez, Carmen Montero Martinez, Carmen Soria Esojo, Carmen Soto Fernandez, Carolina Puchaes Manchon, Carracedo Sevillano Martín, Catalina Balaguer Balaguer, Celia Lacarcel Bautista, César Aurelio Gutiérrez Rivera, Christian Anchorena Diaz, Christian Garcia Fadul, Christian Teijo Núñez, Chunshao Hu Yang, Concepcion Cañete Ramos, Concepción Hernández García, Coral Gonzalez Fernandez, Cristian Avon Gomez, Cristina Benito Bernáldez, Cristina Miralles Saavedra, Cristina Navarro Soriano, Cristina Represas Represas, Cristina Solé Felip, Cristobal Esteban, Damián Malia Alvarado, Daniel Mejías Hidalgo, Daniel Rey Aldana, Daniel-Ross Monserrate Mitchell, Dario Mendez Cabezudo, David Barros Casas, David De La Rosa Carrillo, David Ferrando Garcia, David Iturbe Fernández, David Morchón Simón, David Orts Giménez, Delfin Arzua Mouronte, Demetrio Gonzalez Vergara, Dita Kopecna, Domingo García Aguilar, Eduard Valverde Forcada, Eduardo Arcalá Campillo, Eduardo Marquez, Elena Castro Rodriguez, Elena Molins Millan, Eleuterio Llorca Martínez, Eliana Denise Damonte White, Elisabeth Castillo Diaz, Elsa Naval, Emma Vazquez Espinosa, Emmanuel Coloma Bazán, Enith Yojana Garcia Carrascal, Enric Hernandez Huet, Enrique Cabrerizo Cómitre, Enrique Jesus Alvarez Asensio, Enrique Temes Montes, Erick Monclou Garzón, Esteban Ciruelos Ayuso, Esteban Martin Echevarria, Esther Alonso Sastre, Esther Antón, Esther Arias Alba, Esther Rodriguez González, Eva Belen De Higes Martinez, Eva Cabrera César, Eva Farrero Muñoz, Eva Maria Rosillo Hernandez, Eva Martinez Moragon, Eva Prats, Fco. Javier Ruiz Moruno, Federico Iglesias, Felipe Nicolau Pastrie, Felix Ortiz Portal, Fernando Bonet Madurga, Fernando De Arriba Frade, Fernando Gutierrez Marcos, Fernando Iglesias Rio, Fernando Leon Marrero, Fernando Maria Navarro Ros, Fernando Molina Nieto, Fernando Muñoz Díaz, Fernando Pedraza Serrano, Fernando Sanchez-Toril Lopez, Francesc Puchades, Francisco Astudillo Martin, Francisco Beltrán Baldovi, Francisco Canales Cid, Francisco Casas Maldonado, Francisco De Pablo Cillero, Francisco Espildora Hernández, Francisco Javier Gomez De Terreros Caro, Francisco Javier Gonzalez Barcala, Francisco Linde De Luna, Francisco López García, Francisco Martín Luján, Francisco Ortega Ruiz, Frederic Tatay Soler, Germán Luis Llavador Ros, German Saez Roca, Gonzalo Segrelles Calvo, Graciliano Estrada Trigueros, Gregorio Sanchez Romero, Gustavo De Luiz Martínez, Gustavo Villegas Sanchez, Hemily Katerine Izaguirre Flores, Higinio Vazquez Galdo, Hugo Garcia Ibarra, Hugo Gomez Marquez, Ignacio Garcia-Talavera, Ignacio Javier Pérez De Diego, Ignacio Peñas, Igor Iturbe Susilla, Iker Fernandez-Navamuel, Ingrid Solanes, Inmaculada Salvador Adell, Irene Caselles González, Isaura Parente Lamelas, Ismael Ali Garcia, Ismael Francisco Aomar Millán, Iván Arroyo Fernández, J.Pablo Herrero Jarque, Jaume Joan Ferrer Sancho, Javier Barrio Soto, Javier Elvira Gonzalez, Javier Gallego Borrego, Javier Hueto Pérez De Heredia, Javier Mazo Etxaniz, Javier Morcillo Huertas, Javier Perez Pallares, Jessica Ivana Hilares Vera, Jesus Camino Buey, Jesús Fernandez Frances, Jesus Jimenez Lopez, Jesús Manuel Fernández Villar, Jesús Medina Asensio, Jesús R Hernández Hernández, Jesús Recio Iglesias, Joan Ramon Rozadilla Sacanell, Joan Sola Aznar, Joan Valldeperas, Joaquín Alfonso Megido, Joaquin Cegoñino De Sus, Joel Alejandro Varona Sanchez, Jordi Juanola Pla, Jorge Manuel Romero Requena, Jorge Pascual Bernabeu, Jose Alberto Martos Velasco, Jose Antonio Cascante Rodrigo, José Antonio Castilllo Vizuete, José Antonio Díaz Peromingo, José Antonio Marín Torrado, Jose Arnoldo Grajeda Juarez, Jose Bascuñana Morejon De Giron, Jose Bujalance Zafra, Jose Calvo Bonachera, Jose Carlos Serrano Rebollo, Jose Celdran Gil, Jose Daniel Alcazar Ramirez, José Enrique Gavela García, Jose Francisco Carboneros De La Fuente, José Ignacio De Granda-Orive, José Ignacio Herrero Herrero, José Ignacio Pardo González De Quevedo, José Ignacio Quintana González, Jose Luis Casells Hernandez, José Luis Córcoles Satorre, Jose Luis Delgado Morales, Jose Luis Diez Jarilla, Jose Luis Fernández Sánchez, Jose Luis Gutierrez Alonso, Jose Luis Rojas Box, José Luis Trujillo Castilla, Jose Luis Velasco Garrido, Jose M Marin Trigo, Jose Manuel Garcia Pazos, Jose Manuel Querol Borras, Jose Manuel Vera Aranda, Jose Manuel Villar Freire, Jose Maria Cruz Molina, Jose Martin Clos, Jose Miguel Rodriguez Gonzalez-Moro, José Miguel Seguí Ripoll, Jose Miguel Valero Perez, Jose Ramon Donado Uña, José Ramón Ferrando Gabarda, Jose Vicente Greses Giner, Jose Vicente Puchades Castelló, Jose Vicente Roig Figueroa, Josep Mª Alsina Martí, Juan Manue Diez Piña, Juan Abreu González, Juan Antonio Martinez Carbonell, Juan Antonio Santos Bermejo, Juan B. Bauza Deroulede, Juan Bautista Montagud Moncho, Juan Carlos Tapia Regidor, Juan Francisco De Vega Garcia, Juan Gonzalez Rodriguez, Juan Jose Cruz Rueda, Juan Luengo Alvarez, Juan Luis Rodriguez Hermosa, Juan Manuel Sanchez Apresa, Juan Manuel Verdeguer Miralles, Juan Marco Figueira Gonçalves, Juan Marcos Moreno Mendaña, Juan María Rubio Sánchez, Juan Ortiz De Saracho Bobo, Juan Pablo De Torres Tajes, Juan Pablo Garcia, Julia Garcia De Pedro, Julia Tabara Rodriguez, Julián Andrés Ceballos Gutiérrez, Julio Hernández Vázquez, Justo Grau Delgado, Laura Caballero Ballesteros, Laura Ovejero García, Laura Rodriguez Pons, Laureano Gomez Gonzalez, Leopoldo Damian Dominguez Perez, Leyre Serrano Fernandez, Lourdes Lázaro Asegurado, Lourdes Palomo Alameda, Lucia Diaz Cañaveral, Lucia Gil Maneu, Lucía Zamora Molina, Luis Adolfo Urrelo Cerrón, Luis Alfonso Sota Yoldi, Luis Emilio Planelles Herrero, Luis Fernando Cabañas Enriquez, Luis Fernando Cassini Gómez De Cádiz, Luis García-Giralda Ruiz, Luis Máiz, Luis Manuel Entrenas, Luis Miguel Cuadrado Gomez, Luis Miravet Sorribes, Luis Obradors, Luis Puente Maestu, Luis Rodriguez Pascual, Lyam Lamrini Hamido Laarbi, M Luisa Nieto Cabrera, M. Carmen Gallego Bermejo, Mª Carmen Bermejo Navas, Mª Del Carmen Fernandez Garcia, Mª Lourdes Ramos Casado, Mªcruz Almendros Rivas, Magdalena Serradilla Sánchez, Manuel Arenas Gordillo, Manuel F Ramirez Espinosa, Manuel Jesús Menduiña Guillén, Manuel Jesús Romero Jiménez, Manuel Lorenzo López Reboiro, Manuel Maldonado Escobar, Manuel Martínez Riaza, Manuel Mazabel Flores, Manuel Quirós Valera, Manuel Suárez Tembra, Manuel Tumbeiro Novoa, Manuel Vilà Justribó, Mar Garcia Perez, Marcel Jose Rodriguez Guzman, Mari Carmen Anton Sanz, Maria Belen Marin Martinez, Maria Carme Puy Rión, Maria Carmen Aguar Benito, Maria Carmen Morillo Dominguez, Maria Cruz Gonzalez Villaescusa, Maria De Las Nieves Jimenez Baquero, María Del Carmen García García, Maria Del Carmen Villa Corbaton, Maria Del Mar Bedia Monet, Maria Del Mar Lucena Merino, Maria Del Mar Rodriguez Alvarez, María Del Pilar Martínez Olondris, Maria Del Roser Costa Solà, Maria Dolores Corbacho Abelaira, Maria Dolores Hisado Díaz, Maria E. Barroso Medel, Maria Elia Gómez Merino, Maria Emilia Navascues Martinez, Maria Encarnación Hernández Contreras, Maria Gomez Antunez, María Gómez Peña, María González Gómez, Maria Hernandez Bonaga, Maria Hernández Roca, María Inés Prieto López, Maria Jesus Aviles Ingles, María Jesús Cazorla Martinez, María Jesús Igúzquiza Pellejero, Maria Jesus Linares Asensio, Maria Jesus Mena Rodriguez, María Jesús Moro Alvarez, María José Muñoz Martínez, María José Sánchez Pérez, Maria Luisa Lopez Diaz-Ufano, Maria Martinez Ceres, Maria Milagros Iriberri Pascual, Maria Paz Valiño Lopez, Maria Rafaela Sanchez Perez, María Ruth Herrero Mosquete, Maria Teresa Rodriguez Iglesias, María Torres - Durán, Mariano Rodríguez Porres, Mario Nicolás Albani Pérez, Marta Ballester Canelles, Marta Palop Cervera, Martín Rubianes Gonzalez, Maryam Sidahi Serrano, Maximino González Fernández, Mcarmen Juarez Morales, Mercedes Duffort Falco, Mercedes Palau Benavent, Mercedes Pallero Castillo, Michael Alwakil Olbah, Miguel Angel Ciscar Vilanova, Miguel Angel Franco Campos, Miguel Angel Martin Perez, Miguel Angel Moscardó Orenes, Miguel Angel Racionero Casero, Miguel Angel Rodriguez Rodriguez, Miguel Barrueco Ferrero, Miguel Miller Guerrero, Miguel Roman Rodriguez, Miguel Sanchez Nanclares, Milagros Gonzalez Bejar, Milko Torres De Castro, Mireia Serra Fortuny, Mohamed Kallouchi, Monica Ponce Sanchez, Monica Romero Nieto, Monica Sanchez Garcia, Muriel Sena Gutiérrez, Myriam Calle Rubio, Nagore Blanco Cid, Natividad Quilez Ruiz Rico, Noelia Cubero De Frutos, Noelia Pablos Mateos, Nuria Castejón Pina, Nuria Celorrio Jimenez, Núria Chamorro Tort, Nuria Galofré, Nuria Reina Marfil, Nuria Rodriguez Lázaro, Oscar Bernadich, Pablo Catalán Serra, Pablo Espejo Salamanca, Pablo Rubinstein, Pascual Cortes Miro, Patricia Mata Calderón, Patricia Mínguez Clemente, Patricia Sobradillo Ecenarro, Paula Rodriguez Rodriguez, Pedrito Tomas Tobaruela, Pedro Cancelo Suarez, Pedro J Romero Palacios, Pedro Jose Cordero Rodriguez, Pedro Landete Rodriguez, Pedro Luis Baños Hidalgo, Pedro Penela Penela, Pere Almagro Mena, Pere Serra Mitjà, Pilar Gil Alaña, Pilar Gispert Del Rio, Pilar Iñigo Naranjo, Pilar Marin Martinez, Póvilas Gerardo Dambrava Rodríguez, Rafael Castillo Rubio, Rafael Castrodeza Sanz, Rafael Garcia Carretero, Rafael Lama Martinez, Ramon Boixeda Viu, Ramon Magarolas Jordà, Raquel Dacal Quintas, Raquel García Sevila, Raquel Morillo Guerrero, Raquel Rodriguez Martínez, Raquel Sánchez Juez, Raul Sandoval Contreras, Ricardo Diaz-Casteleiro Romero, Ricardo Ismael Aguilar Perez Grovas, Roberto Bernabeu Mora, Roberto Chalela Rengifo, Roberto Gorgues Comas, Roberto Hurtado Garcia, Roberto Ordóñez Picón, Rocio García García, Rodolfo Álvarez-Sala, Romualdo Roncero Vidal, Rosa Malo De Molina Ruiz, Rosa Maria Folgado De La Fuente, Rosa Maria López Lisbona, Rosa Nieto, Rosario Martinez Barea, Rossana Isabel Baron Lopez, Ruben Lera Alvarez, Salud Santos Pérez, Salvador Alvarez Martin, Salvador Bello Dronda, Salvador Díaz Lobato, Santiago Balmes Estrada, Santiago Carrizo Sierra, Santiago Rubio - Félix, Segismundo Solano Reina, Sergio Alcolea Batres, Sergio Cinza Sanjurjo, Sergio Curi Chercoles, Sergio Fandos Lorente, Sergio Manuel Marti Beltran, Silvia Fernandez Huerga, Silvia Merlos Navarro, Silvia Pérez Fernández, Silvia Sánchez, Sladana Obradovic, Soledad Alonso Viteri, Sonia Martínez Sáez, Susana Filgueira Martínez, Tamara Alonso Pérez, Teresa Peña Migul, Tomas De Vega Santos, Uan Ignacio Ramos Clemente Romero, Vanessa Zorrilla Lorenzo, Vicenta Cresencio Perez, Vicente Quinzá Valero, Victor Gaya Sancho, Victor Manuel Mora Cuesta, Victoria Valeri-Busto Gonzalez, Virginia Almadana Pacheco, Walther Ivan Giron Matute, Xavier Flor, Xavier Pomares Amigó, Xavier Vila Giralte, Xoel Pena Perez, Yadira Dobarganes Sansón, Yoana Lazaro Salazar, Yussef Abu El Wafa and Zully Vasquez Gambasica. All of these authors mentioned collected data.
